# Mid-IR Hollow-core microstructured fiber drawn from a 3D printed PETG preform

**DOI:** 10.1038/s41598-018-26561-8

**Published:** 2018-05-25

**Authors:** Wanvisa Talataisong, Rand Ismaeel, Thiago H. R. Marques, Seyedmohammad Abokhamis Mousavi, Martynas Beresna, M. A. Gouveia, Seyed Reza Sandoghchi, Timothy Lee, Cristiano M. B. Cordeiro, Gilberto Brambilla

**Affiliations:** 1 Optoelectronics Research Centre, University of Southampton, Southampton, SO17 1BJ UK; 2 Instituto de Fisica “Gleb Wataghin”, Universidade Estadual de Campinas (UNICAMP), Campinas, Brazil; 3 Instituto de Filosofia e Ciências Humanas, UNICAMP, Campinas, Brazil

## Abstract

Mid-infrared (mid-IR) optical fibers have long attracted great interest due to their wide range of applications in security, biology and chemical sensing. Traditionally, research was directed towards materials with low absorption in the mid-IR region, such as chalcogenides, which are difficult to manipulate and often contain highly toxic elements. In this paper, we demonstrate a Polyethylene Terephthalate Glycol (PETG) hollow-core fiber (HCF) with guiding properties in the mid-IR. Guiding is provided by the fiber geometry, as PETG exhibits a material attenuation 2 orders of magnitude larger than the HCF propagation loss. The structured plastic fiber preforms were fabricated using commercial 3D printing technology and then drawn using a conventional fiber drawing tower. The final PETG fiber outer diameter was 466 µm with a hollow-core diameter of 225 µm. Thermal imaging at the fiber facet performed within the wavelength range 3.5–5 µm clearly indicates air guidance in the fiber hollow-core.

## Introduction

Since the invention of laser sources in the mid-IR range spectral region (2.5 to 25 μm), there has been a growing interest in the development of optical fibers transparent at these wavelengths for applications in chemical, biological and atmospheric sensing^1–4^, where the unique molecular absorption associated to the excitation of specific fundamental vibrational and rotational modes allows for an accurate chemical fingerprinting^5^.

Although over the last 3 decades silica optical fibers have become the backbone of optical communications and distributed optical sensing, they cannot guide light in the mid-IR as the silica network has strong overtone absorptions above 2 μm, resulting in an overall attenuation that increases for increasing wavelengths. Thus, the search for optical fiber operating in the mid-IR has focused on materials that have high transmittance in the wavelength range λ = 2–20 μm such as chalcogenide glasses, heavy metal fluorides, polycrystalline silver halides (AgX), single-crystal sapphire, and tellurium halides (TeX)^6–8^, which still requires extraordinary purification processes and are extremely hard to handle.

An alternative approach relies on the use of hollow-core fibers (HCFs) such as photonic bandgap fibers and anti-resonant fibers, where light is confined within the hollow-core, greatly decreasing the influence of the material optical properties. Such fibers have been already exploited for numerous applications in infrared spectroscopy such as laser surgery, gas sensing, label-free biological sensing, thermal imaging and infrared countermeasures^9–13^. In initial attempts, mid-IR HCFs have been fabricated by coating the inner surface of a capillary tube with a metallic film and dielectric material. However, the coating procedure is relatively cumbersome and used toxic materials. Lately, pure silica HCFs have been investigated^14^. As the mode propagates in the microstructured fiber hollow-core, the effect of the silica’s high material absorption is minimized owing to the low overlap (<1%) of the guided mode with the glass. Silica HCFs were manufactured using the stack and draw technique, where capillaries are manually assembled into a hexagonal structure prior to draw. Despite this being the most popular method, it is labor intensive, mainly limited to the hexagonal packed periodic structure and difficult to implement for complex designs.

Because of the continuous improvement in additive manufacturing technologies and the related cost reduction, the fabrication of prototypes using 3D printing has gained interest in many applications such as medical, art, engineering and science. The most common manufacturing techniques to print 3D models include photo-polymerization, selective laser sintering (SLS), continuous liquid-interface production (CLIP), and fused-deposition modeling (FDM). In photo-polymerization, lasers or light-emitting diodes (LEDs) are scanned across a plane and polymerization occurs where light interacts with the monomer. To achieve 3D profiles, the laser or LED is fixed to a three-axis stage and the process is repeated layer by layer. SLS is one of the 3D printing techniques that can be used to print not only plastic materials but also glass, metal, and ceramic powders. In SLS, a high-power laser is scanned on the horizontal plane layer-by-layer whilst the printing bed is lowered. Under laser irradiation the printing material fuses forming the desired 3D geometry. FDM is the most commonly used (and cheapest) technique and relies on a polymer filament fed through a heated nozzle oozing molten polymer. The 3D geometry is generated repeating iteratively the process layer-by-layer.

In photonics, 3D printing has been used to manufacture waveguides and HCFs for terahertz applications^15^. Since devices operating in the terahertz region require dimensions comparable to the width of the 3D printed structures, HCFs were directly 3D printed without any subsequent drawing^16–18^. Simultaneously, the fabrication of solid core optical fibers drawn from 3D printed preforms has been investigated^19^. The microstructured optical fiber preform was printed using a 3D printer operating with the FDM technique and then drawn into an optical fiber on a drawing tower equipped with a low-temperature furnace. A step-index plastic fiber has been drawn in 2016^20^ from a preform printed using a two-nozzle 3D printer with Acrylonitrile Butadiene Styrene (ABS) and Polyethylene Terephthalate Glycol (PETG) filaments. This fiber revealed the possibility of guiding light at telecom wavelengths. The idea of 3D printed hollow-core fiber preform firstly emerged in 2016^21^, where the authors presented the potential of drawing a fiber cane from a 3D printed HCF preform by using a PMMA filament. In 2017, the first drawn hollow-core fiber cane based on a 3D printed preform was demonstrated^22^, but no guiding was observed. Both circular and rectangular hollow-core fiber preforms were 3D printed using ABS filaments, but neither resulted in a HCF. The use of 3D printing has the potential to fabricate fibers with arbitrary shape^23,24^ that cannot otherwise be realized using drilling/machining or the stack-and-draw technique, thus immensely expanding the design capabilities.

Here, we demonstrate a HCF drawn from a 3D printed fiber preform, capable of guiding light in the mid-IR. The FDM printing method is chosen to print a hollow-core preform using a transparent PETG filament. Despite PETG having a bulk material absorption in excess of 10 dB/mm in the 3.5–5 μm range, light is guided using the antiresonance confinement resulting in a fiber with a propagation loss two orders of magnitude smaller than the PETG absorption loss.

## 3D Printing and Drawing

**Figure 1:**
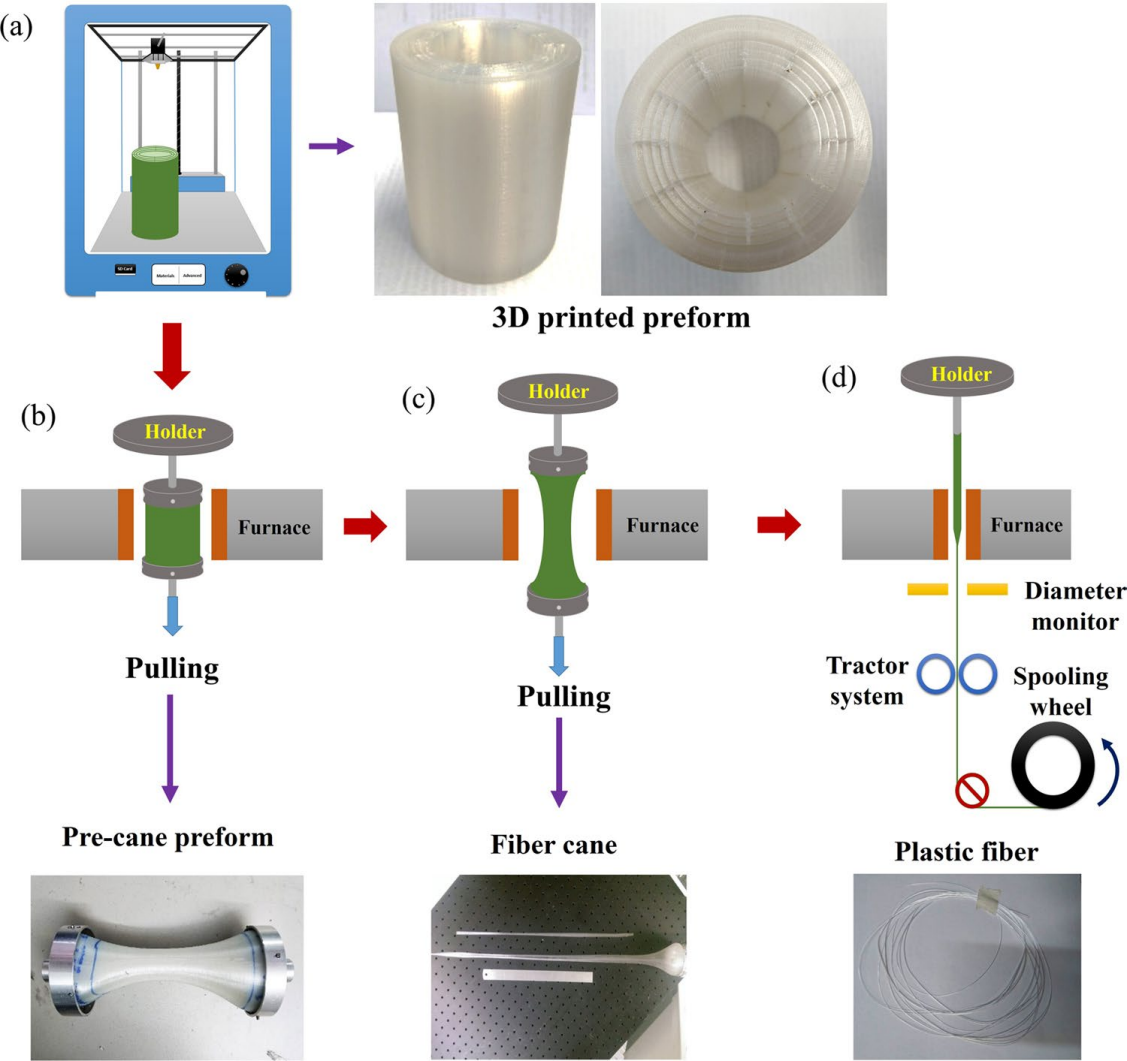
(**a**) Hollow-core fiber preform printed using a commercial 3D printer. (**b**) High temperature pre-caning to reduce the preform size followed by (**c**) cane drawing and (**d**) fiber drawing.

3D models of hollow-core preform were designed using the “Fusion 360” software (Autodesk) and realized as the preform of Fig. 1(a) as explained in the methods section. Although the polymer filament used to print the fiber preform has optical quality, thus is visually transparent, the 3D printed prototype exhibits a significant scattering (thus a milky color), arising from air bubbles trapped in the preform during the printing process. The 3D printed preform is constructed by depositing the melted polymer filament layer-by-layer and any air trapped in the printed interfacial layers compromises the interlayer adhesion. Additionally, bubbles expand when the preform is heated resulting in a significant deformation of the drawn fiber, delamination of the fiber preform layers and a sudden preform breakage. The preform transparency was therefore optimized by ensuring that adjacent printed lines fused without any air trapping as explained in the methods section.

Fiber drawing from the 3D printed preform is divided into three stages: (1) the 3D printed preform (Fig. 1(b)) is tapered into a pre-cane preform with a ~40 mm diameter; (2) the pre-cane preform is drawn into a ~15 mm diameter cane (Figs 1(c) and (3)) the fiber is drawn from the cane (Fig. 1(d)).

**Figure 2:**
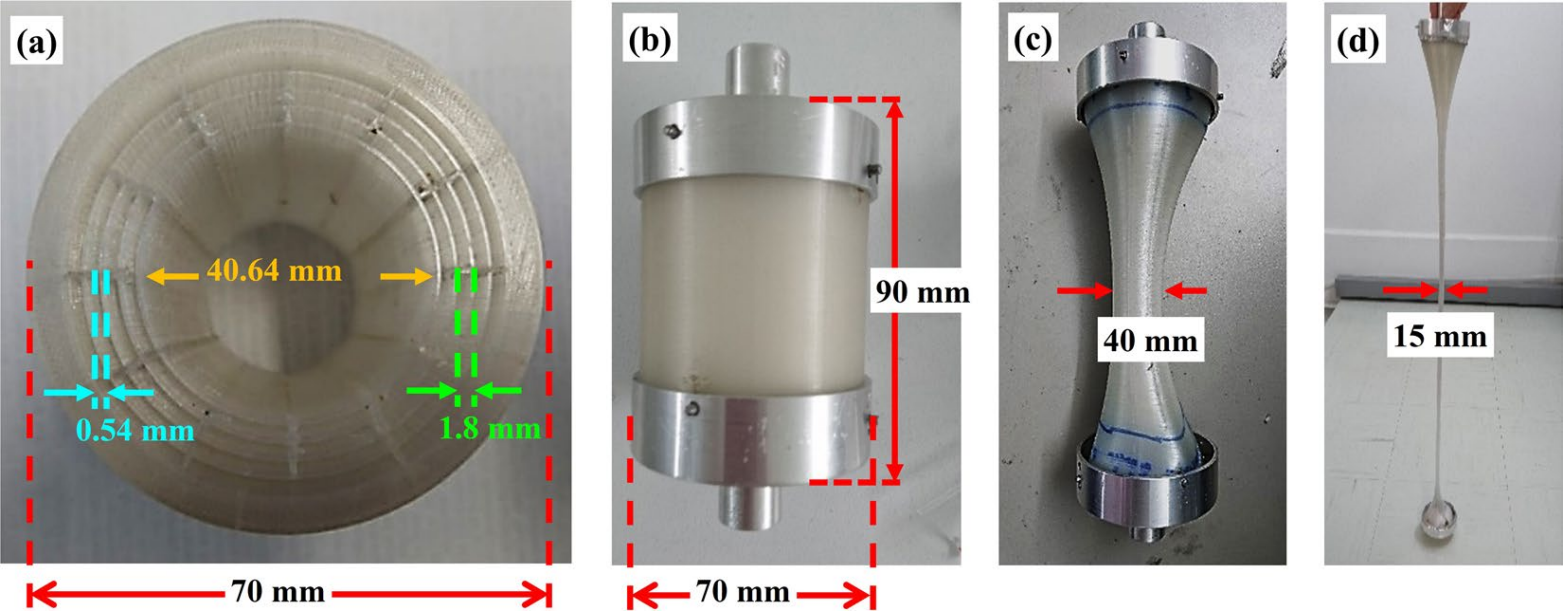
(**a**) Cross-section of the 3D printed hollow-core PETG fiber preform with related dimensions. (**b**) Assembly of the 3D printed fiber preform with the holders prior to pre-caning. (**c**) Pre-cane preform. (**d**) Fiber cane.

**Figure 3:**
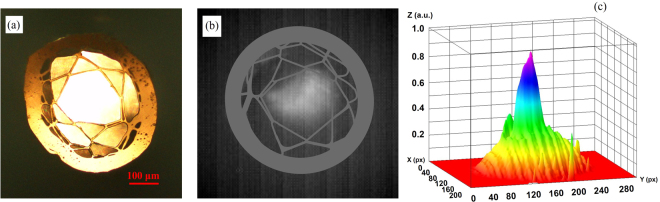
(**a**) Hollow-core fiber cross-section under an optical microscope operating in the visible. (**b**) Mid-IR mode image and (**c**) Intensity profile.

In the first stage, the top and the bottom parts of the hollow-core fiber preform (Fig. 2(a)) were inserted into specifically designed holders (Fig. 2(b)) to minimize changes in the fiber structure during drawing^25,26^ in a manner similar to that used in the fabrication of PMMA fibers. The top holder was connected to an XYZ translation stage used to control the position of the preform and its feeding speed into the furnace. A ~90 mm bore radiative furnace was used to anneal the preform at T = 150 °C before pulling. The bottom holder moved at a pulling speed of 0.01 m/min while the preform was kept at T = 150 °C which produced a ~40 mm diameter pre-cane preform (Fig. 2(c)). The annealing and pulling processes were repeated at T = 150 °C and delivered a cane with a final diameter of ~15 mm (Fig. 2(d)). The cane was heated by using a smaller furnace with a 25 mm bore to match the smaller size of the cane and pulled into the hollow-core fiber without any pressurization being applied. The PETG viscosity at this processing temperature was estimated to be above 10^5^ Poise, providing an operational regime where viscosity dominates on surface tension, avoiding interstice closure. The HCF was cleaved by using a heated razor blade and optical microscope images of its cross-section are presented in Fig. 3. Although the fiber preform 3D printing parameters such as infill density and printing speed as well as additional preform pre-annealing were optimized to minimize air trapping, some air bubbles still survived and can clearly be seen in the fiber cladding (Fig. 3(a)). The final optical fiber exhibited a slightly elliptical cross-section, which was ascribed to the asymmetry introduced by the longitudinally layer-by-layer preform printing and by the small degree of swelling associated to air and moisture trapped in the polymeric matrix during printing. The fiber diameter was measured to be ${\varnothing }_{{\rm{OD}}1}$ ~ 466 μm and ${\varnothing }_{{\rm{OD}}2}$ ~ 417 μm along the major and minor axes, respectively. The core (hole) diameters for the major and minor axes were measured to be ${\varnothing }_{{\rm{c}}1}$ ~ 225 μm and ${\varnothing }_{{\rm{c}}2}$ ~ 177 μm, respectively.

## Results and Discussion

In order to evaluate the optical guidance properties of the hollow-core fiber in the mid-IR, a broadband lamp with emission in the wavelength range 450–5500 nm (Thorlabs SLS202) was launched into a 12 cm long section of fiber using a bare-fiber adapter. The modal image was taken by collecting the fiber output in the wavelength range λ = 3.5–5 μm into a thermal infrared camera (Onca-MWIR-Insb) using a ZnSe objective lens with an 18 mm focal length. Figure 3 presents the thermal image and its intensity profile at the HCF output. Figure 3(b) shows that mid-IR light is guided in the air-core, and modeling ascribed this to antiresonant reflection at the first layer of polymer strands (Fig. 3(b,c)).

**Figure 4:**
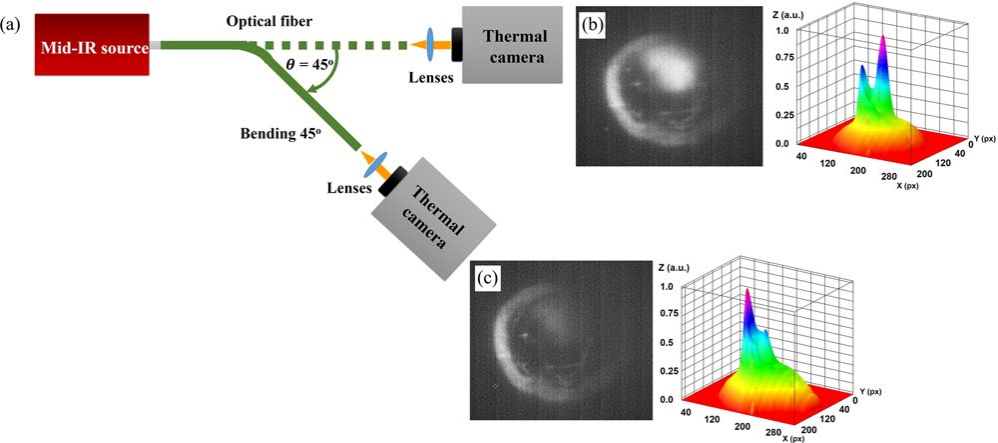
(**a**) Schematic diagram of the experimental setup used for the mid-IR modal imaging from the HCF during bending. (**b**) Modal image and intensity profile of light transmitted through the air-core fiber when straight, and (**c**) Experienced a 45° bending.

To confirm the HCF mid-IR guiding capability, the near-field image of the transmitted light in the wavelength range λ ~ 3.5–5 μm from the fiber facet was recorded under different degrees of fiber bending, as shown in Fig. 4(a). The bending radius varied from 0 to 45° with the radius of curvature of ~76 mm. Figure 4(b,c) show the HCF output and its intensity profile for the straight and bent cases. While in the straight fiber most of the light is guided in the air-core (Fig. 4(b)), when the fiber is bent, an increasingly larger amount of light is coupled to the polymer cladding (Fig. 4(c)). The different intensity profiles of Fig. 3(c) and 4(b) are caused by the different fiber cleaving. Cleaving is the main reason for the deformation of the microstructured optical fibers, which results in different light launching conditions, thus different intensity profiles at the fiber output.

**Figure 5:**
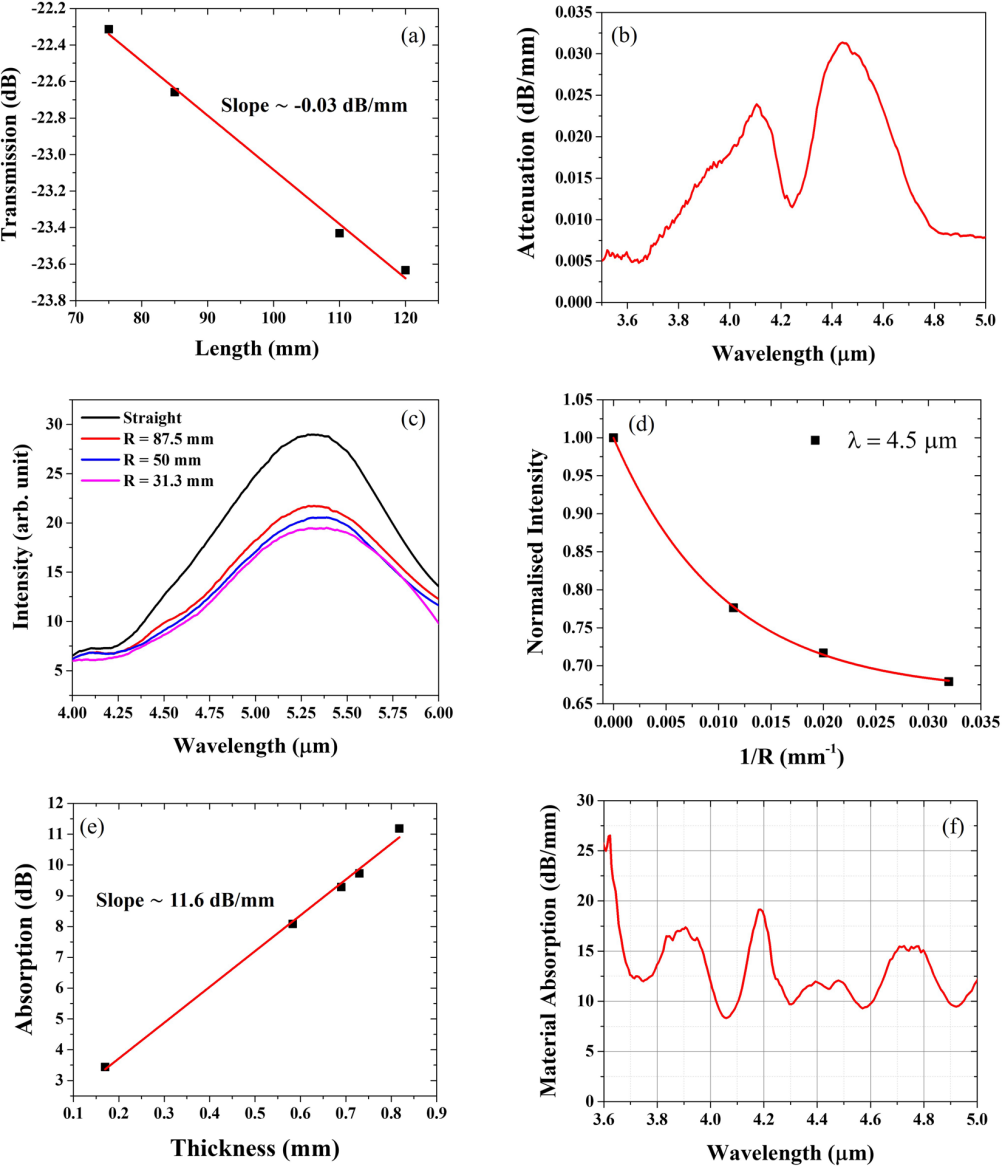
(**a**) Relationship between transmission at λ ~ 4.5 µm and fiber length. (**b**) Spectral attenuation of the optical fiber; the low loss at λ < 3.55 µm is an artefact resulting from the small signal to noise ratio in the transmission measurements. (**c**) Fiber transmission spectra obtained in the straight fiber and at bend radii of 31.3, 50, and 87.5 mm. (**d**) Normalized transmitted intensity at different bend radii at the wavelength of 4.5 µm; the red line represents a polynomial fit. (**e**) Relationship between PETG absorption and sample thickness at λ ~ 4.5 µm. (**f**) Plot of PETG material absorption versus wavelength.

The cut-back measurement was used to determine the propagation loss. In this setup, mid-IR light was launched into the hollow-core fiber from a broadband visible to mid-IR lamp (Thorlabs SLS202). The transmission spectra at different fiber lengths (75, 85, 110 and 120 mm) were measured using a FTIR spectrometer (ARCspectro FT-MIR) and confirmed a propagation loss of 0.03 dB/mm at λ ~ 4.5 µm (Fig. 5(a,b)). Bend measurements were performed with different bend radii of 31.3, 50, and 87.5 mm. As before, light from a broadband visible to mid-IR lamp was launched into a hollow-core fiber and the transmission spectra of the optical fiber with different bend radii were measured using a FTIR spectrometer. From the experimental results, the bend loss at λ ~ 4.5 µm for the bend radius of 31.3 mm is 17 dB/m, as presented in Fig. 5(c). The material absorption of the PETG filament used to print the fiber preform was also measured using the cut-back technique by launching light through the side of planar filaments with different thicknesses. The FTIR spectrometer was used to measure the transmission spectra of different sample thicknesses, showing that the material absorption of PETG at λ ~ 4.5 μm is 11.6 dB/mm (Fig. 5(e)), 2 orders of magnitude higher than the HCF total attenuation. This cut-back process was then applied to the whole wavelength range 3.5–5 µm, providing an average material attenuation in excess of 8 dB/mm over the whole spectral region reported in Fig. 5(f). Yet, mid-IR light can be guided in the air-core of the fabricated optical fiber over the length of 10 cm.

**Figure 6:**
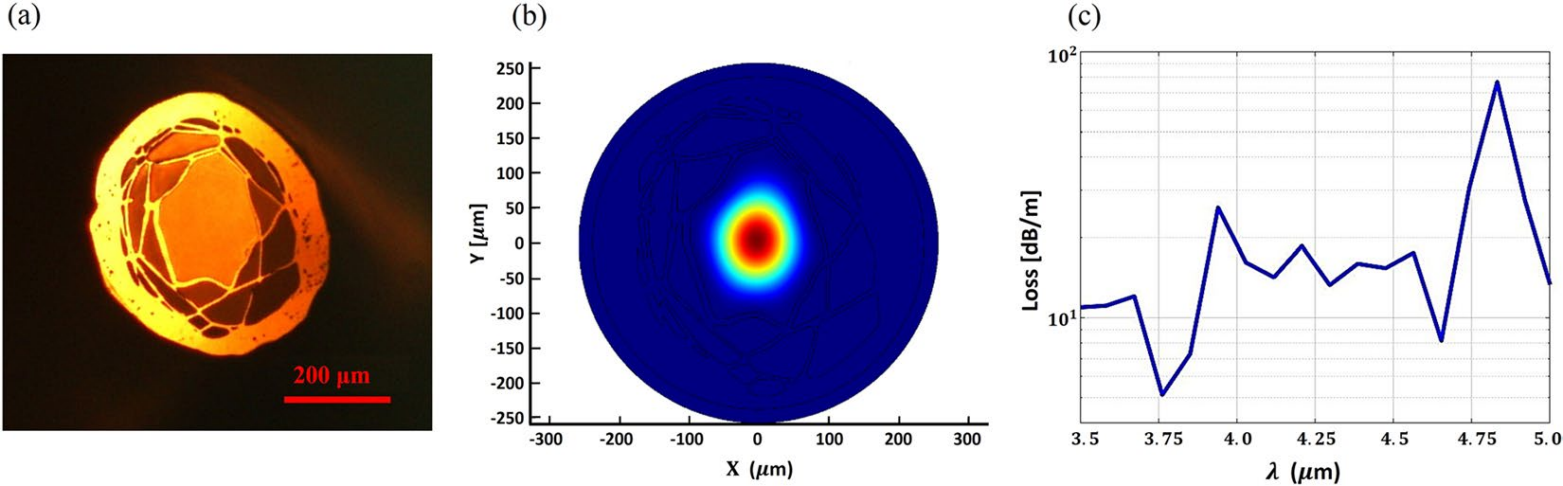
(**a**) Microscope image of the fiber cross-section used in the modal simulations, (**b**) Intensity profile of the mode supported by the air-core in the simulated fiber structure at λ ~ 4.5 mm and (**c**) simulated propagation loss in the wavelength range λ = 3.5–5 μm taking into account material absorption loss.

Simulations of the mode propagating through the HCF structure were performed by importing into a commercial finite element method software (COMSOL^©^) the cross-section microscope image (Fig. 6(a)) of the fiber used for loss measurements. Refractive index of material used in the simulation was obtained from the graph of refractive index of Polyethylene Terephthalate film with the wavelength^27^. Figure 6(b) shows the supported mode, confined in the air-core at λ ~ 4.5 μm. The propagation loss was also calculated using the same HCF profile and importing the PETG absorption measured previously on the filament bulk sample. The resulting loss profile (Fig. 6(c)) shows that attenuation is lower at shorter wavelengths and it increases at longer wavelengths and it is generally between 1 dB/m and 100 dB/m. This is expected, as the optical mode spreads further from the hollow-core at longer wavelengths, resulting in larger overlaps with the polymer material, thus larger losses. The high loss measured in the manufactured HCF has been attributed to the irregular fiber profile along the azimuthal direction, an assumption supported by the different cross section profiles observed during the fiber cleaving (see Figs 3(a) and 6(a)).

## Conclusions

In conclusion, a polymer air-core optical fiber fabricated from a 3D printed preform is demonstrated. Although the final structure of the fiber experienced a strong deformation during the drawing process, guidance in antiresonant fibers is provided by the innermost layers, thus strong cladding deformations are relatively insignificant: transmission in the mid-IR wavelength region (λ ~ 3.5–5 μm) over a 10 cm long fiber was observed. The mid-IR near-field end facet image and the modal profile simulations show the ability to confine mid-IR light in the air-core. The calculated propagation loss, including the material loss, reveals a potential loss of 20 dB/m at *λ *~ 4 μm. The measured propagation loss, (of the order of 30 dB/m) is 2 orders of magnitude smaller than the polymer loss and can be improved by reducing the structural deformations associated to printing and fiber drawing.

## Methods

The HCF preform in this experiment consists of a circular air-core surrounded by 4 periodic layers of a polymer cladding spaced by air gaps. Light in the hollow-core fiber can be confined to the central air-core owing to the Bragg reflection from the periodic cladding structure. This structure was chosen because its hollow- core gives easy access to fluidic and gaseous samples, which then have a strong overlap with the optical field. Optical fibers based on this cobweb cladding structure do not require small (e.g. 2–10 µm) hollow-cores to guide light, as in other microstructured fibers; above all, they provide a good mechanical stability during drawing. In the design, the preform length (*L*) and diameter ($\varphi$) were chosen to be *L* = 90 mm and $\varphi$ = 70 mm, to fit the furnace bore. The thickness of the polymer cladding layers was chosen to be 0.54 mm with a 1.8 mm air-gap between them. The bridges connecting each cladding polymer layer were also 0.54 mm thick. The design of similar hollow- core fiber structure has been reported in 2006^28^.

The optical fiber preforms were printed by feeding PETG in a commercial FDM thermal 3D printer (Ultimaker 2 extended+). PETG was chosen because of its strength and durability and its relatively small production of fumes when printed and drawn compared with other polymers (such as acrylonitrile butadiene styrene - ABS).

The preform transparency was strongly affected by extruder temperature, material flow rate, and infill pattern. PETG viscosity has a weak dependence on the temperature and common processing temperatures can be in the range T = 180 °C to 295 °C^29^ as they strongly depends on additives, average polymer molecular weight or processing speed. Here, the 3D printer extruder working temperature was ranged from 230 °C to 255 °C in steps of 5 °C to find the optimal processing conditions. At the low temperatures, the filament thread had high viscosity and poor layer to layer adhesion, resulting in easy delamination during fiber drawing. The transparency of the 3D printed prototype improved when the temperature was increased to 250 °C, but at higher temperatures (T > 255 °C), the printed objects started to exhibit a yellow coloration attributed to the polymer degradation as a result of overheating. This can ultimately lead to the nozzle blockage and thus the extruder temperature was set to 250 °C.

**Figure 7:**
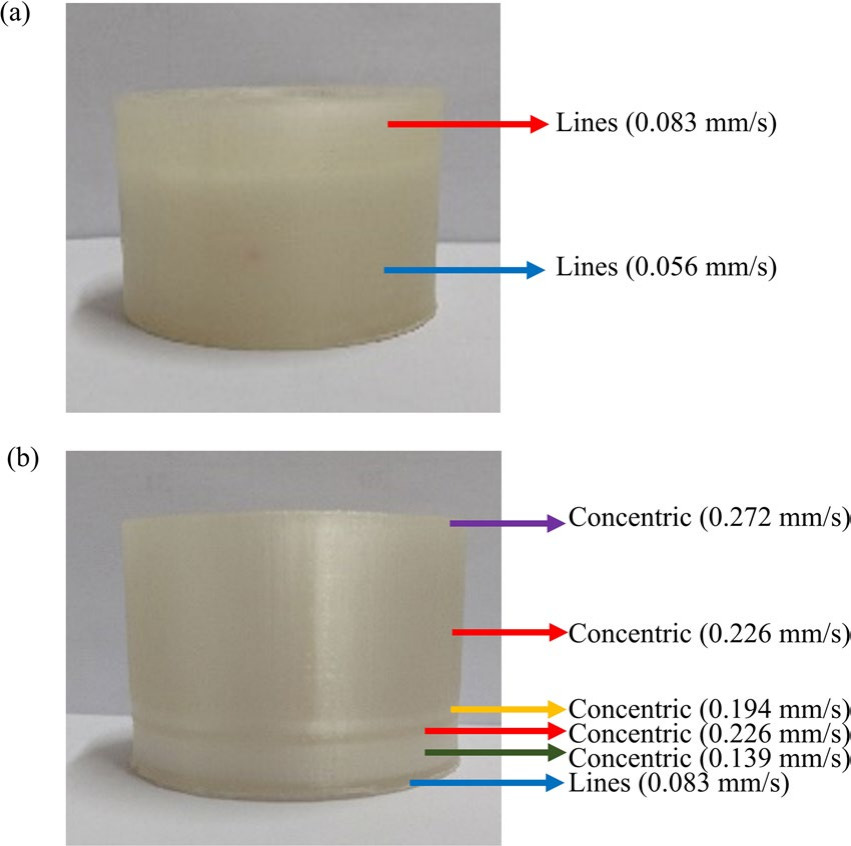
Dependence of printed preform transparency on the material flow rate with (**a**) lines and (**b**) concentric infill patterns.

The effect of material flow rate and infill pattern on transparency were also investigated. The default material flow rate of the printer was measured to be 0.056 mm/s. At this flow rate, the material deposited by the extruder does not fill all voids resulting in a relatively large light scattering and low transparency. As shown in Fig. 7(a), the adoption of a line infill pattern and an increase of the material flow rate to >0.1 mm/s allowed a transparent preform to be achieved (Fig. 7 (a)). Different infill patterns were investigated: while parallel lines patterns have shown good results for prototypes with parallelogram like shapes, the concentric infill pattern was found to be best suited for cylindrical structures, providing a superior layer to layer adhesion, lower anisotropy and smaller deformations during pulling. Additionally, the adoption of a concentric pattern allowed for the reduction of the preform printing time by 6 hours (from 92 to 86 hours) with respect to the line infill pattern.

The printing flow rate for the concentric infill pattern was then optimized for transparency, as illustrated in Fig. 7(b). When using the same material flow rate, the printed preform section with concentric infill pattern exhibits larger scattering (thus looks less transparent) than the line infill pattern section. Yet, lower scattering, thus larger transparency with the concentric infill pattern occurred when the material flow rate increased to values in excess ~0.22 mm/s. The preform section printed with 0.272 mm/s flow rate was transparent but exhibited a rough lateral surface due to the excess of material. Because of this, a 0.22 mm/s flow rate for printing with concentric infill pattern was selected.

The deposition layer thickness also influenced the overall preform transparency and it was found that thicker layers provide larger scattering than thinner layers, possibly because of the limited PETG viscosity which does not allow for all gaps to be filled in thick layers. In this work, a layer height of 0.06 mm was chosen as it is the thinnest layer height available with the printer. Finally, the preform transparency was also affected by the bed temperature. While higher bed temperatures are associated with larger transparencies because of the decreased polymer viscosity, it is also associated with larger deformations over long time spans. The bed was fixed at 90 °C for printing PETG.

**Figure 8:**
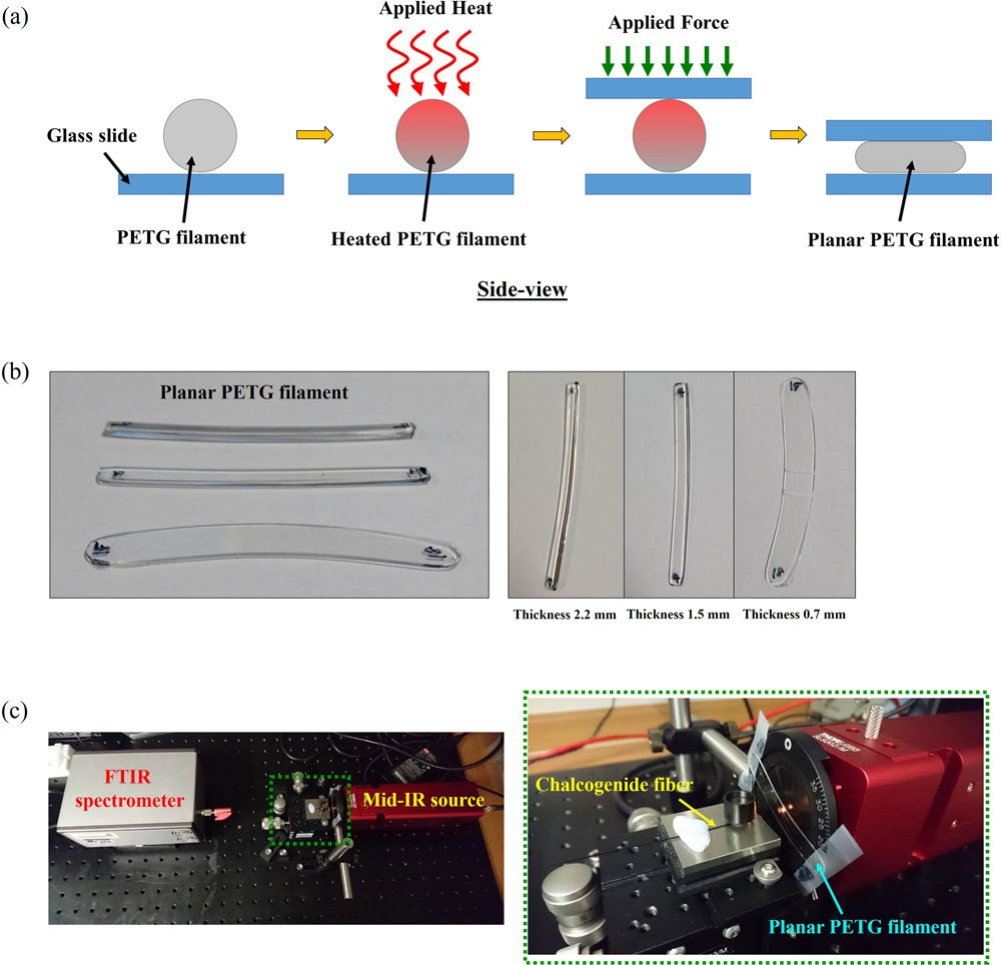
(**a**) Schematic of side-view of experiment setup used to fabricate the planar PETG filament. (**b**) Different thickness–bulk planar PETG samples for material absorption measurement (**c**) Experimental setup used to measure material absorption of a PETG filament in the wavelength range λ ~ 3.5–5 µm.

In this work, the material attenuation of the filament used to print the fiber preform (PETG) was measured. The experimental setup for the material absorption measurement is shown in Fig. 8(c). To analyse the material absorption, the transmission spectra of light propagating through a planar PETG filaments with different thicknesses were measured using the cut-back measurement.

To minimise the measurement error due to reflection and scattering on the standard cylindrical shape filament, the planar PETG filament was created as presented in Fig. 8(a). A short piece of a filament (length ~ 6 cm) was placed on top of a glass microscope slide before being heated to T ~ 180 °C using a hot air gun to ensure uniformity in the temperature profile. The filament was heated until its viscosity decreased, then a second glass slide was placed on the top of the heated filament and force was applied to the top glass slide to flatten the polymeric filament. The thickness of the flat filament was controlled by optimising the force applied on the heated filament. Images of flat PETG samples with different thicknesses are presented in Fig. 8(b).

Light from a broadband visible to mid-IR lamp was launched through the PETG sample at normal incidence and a FTIR spectrometer (ARCspectro FT-MIR) was used to measure the transmission spectra of samples with different thicknesses. A chalcogenide fiber was used to collect light from the source (Thorlabs SLS202) and compared to the light transmitted through the samples.

## References

[1] Brown CW, Chen C-S, Li Y (1995). Near- and mid-infrared chemical and biological sensors. SPIE’s 1995 International Symposium on Optical Science, Engineering, and Instrumentation.

[2] Jha A (2009). Recent advances in mid-IR optical fibres for chemical and biological sensing in the 2–15&mu;m spectral range. Photonics North 2009.

[3] Martínez J (2016). Mid-infrared surface plasmon polariton chemical sensing on fiber-coupled ITO coated glass. Opt. Lett..

[4] Starecki F (2015). Mid-IR optical sensor for CO2 detection based on fluorescence absorbance of Dy3+:Ga5Ge20Sb10S65 fibers. Sensors and Actuators B: Chemical.

[5] Mizaikoff B (2013). Waveguide-enhanced mid-infrared chem/bio sensors. Chemical Society Reviews.

[6] Bureau, B. *et al*. Chalcogenide optical fibers for mid-infrared sensing. **53**, 8 (2014).

[7] Sanghera JS, Shaw LB, Aggarwal ID (2002). Applications of chalcogenide glass optical fibers. Comptes Rendus Chimie.

[8] Tao G (2015). Infrared fibers. Adv. Opt. Photon..

[9] Harrington JA (2000). A Review of IR Transmitting, Hollow Waveguides. Fiber and Integrated Optics.

[10] Kriesel JM (2011). Hollow core fiber optics for mid-wave and long-wave infrared spectroscopy. SPIE Defense, Security, and Sensing.

[11] Patimisco P (2013). Low-Loss Hollow Waveguide Fibers for Mid-Infrared Quantum Cascade Laser Sensing Applications. Sensors (Basel, Switzerland).

[12] Sampaolo A (2015). Single mode operation with mid-IR hollow fibers in the range 5.1-10.5 µm. Opt. Express.

[13] Spagnolo V (2013). Mid-infrared fiber-coupled QCL-QEPAS sensor. Applied Physics B.

[14] Shephard JD (2005). Single-mode mid-IR guidance in a hollow-core photonic crystal fiber. Opt. Express.

[15] Cruz, A. L. S., Argyros, A., Tang, X., Cordeiro, C. M. B. & Franco, M. A. R. 3D-printed terahertz Bragg fiber. *2015 40th International Conference on Infrared*, *Millimeter*, *and Terahertz waves (IRMMW-THz)*, 1-2 (2015).

[16] Cruz ALS, Serrao V, Barbosa CL, Franco MAR (2015). 3D Printed Hollow Core Fiber with Negative Curvature for Terahertz Applications. Journal of Microwaves, Optoelectronics and Electromagnetic Applications.

[17] Li J, Nallappan K, Guerboukha H, Skorobogatiy M (2017). 3D printed hollow core terahertz Bragg waveguides with defect layers for surface sensing applications. Opt. Express.

[18] Ma, T., Nallapan, K., Guerboukha, H. & Skorobogatiy, M. Metallized 3D printed hollow core waveguide Bragg grating for dispersion compensation in terahertz range. *2017 42nd International Conference on Infrared*, *Millimeter*, *and Terahertz Waves (IRMMW-THz)*, 1-1 (2017).

[19] Cook K (2015). Air-structured optical fiber drawn from a 3D-printed preform. Opt. Lett..

[20] Cook K (2016). Step-index optical fiber drawn from 3D printed preforms. Opt. Lett..

[21] Zubel, M. G., *et al*. 3D-printed PMMA Preform for Hollow-core POF Drawing. *The 25th International Conference on Plastic Optical Fibers 2016* (2016).

[22] Marques, T. H. R., Lima, B. M., Osório, J. H., Silva, L. E. D. & Cordeiro, C. M. D. B. 3D Printed Microstructured Optical Fibers. *International Microwave and Optoelectronics Conference* (2017).

[23] Weidenbach M (2016). 3D printed dielectric rectangular waveguides, splitters and couplers for 120 GHz. Opt. Express.

[24] Zhao Q (2017). Optical fibers with special shaped cores drawn from 3D printed preforms. Optik - International Journal for Light and Electron Optics.

[25] Xue SC (2005). Fabrication of microstructured optical fibers-part I: problem formulation and numerical modeling of transient draw process. Journal of Lightwave Technology.

[26] Xue SC (2005). Fabrication of Microstructured Optical Fibers-Part II: Numerical Modeling of Steady-State Draw Process. Journal of Lightwave Technology.

[27] Martínez-Antón JC, Bernabeu E (2002). High performance Feussner-type polarizers based on stretched poly(ethylene-terephthalate) films. Applied Physics Letters.

[28] Huo L, Yu R-J, Zhang B, Chen M-Y, Li B-X (2006). Design Guideline of Hollow-Core Fibres with Cobweb Cladding Structure. Chinese Physics Letters.

[29] O*verview of materials for PETG Copolyester*, http://www.matweb.com/search/DataSheet.aspx?MatGUID=4de1c85bb946406a86c52b688e3810d0.

